# Electron-beam induced deposition and autocatalytic decomposition of Co(CO)_3_NO

**DOI:** 10.3762/bjnano.5.129

**Published:** 2014-07-30

**Authors:** Florian Vollnhals, Martin Drost, Fan Tu, Esther Carrasco, Andreas Späth, Rainer H Fink, Hans-Peter Steinrück, Hubertus Marbach

**Affiliations:** 1 Lehrstuhl für Physikalische Chemie II and Interdisciplinary Center for Molecular Materials (ICMM), Friedrich-Alexander-Universität Erlangen-Nürnberg, Egerlandstr. 3, 91058 Erlangen, Germany

**Keywords:** autocatalytic growth, cobalt tricarbonyl nitrosyl, electron-beam induced deposition, nanofabrication, scanning transmission X-ray microscopy

## Abstract

The autocatalytic growth of arbitrarily shaped nanostructures fabricated by electron beam-induced deposition (EBID) and electron beam-induced surface activation (EBISA) is studied for two precursors: iron pentacarbonyl, Fe(CO)_5_, and cobalt tricarbonyl nitrosyl, Co(CO)_3_NO. Different deposits are prepared on silicon nitride membranes and silicon wafers under ultrahigh vacuum conditions, and are studied by scanning electron microscopy (SEM) and scanning transmission X-ray microscopy (STXM), including near edge X-ray absorption fine structure (NEXAFS) spectroscopy. It has previously been shown that Fe(CO)_5_ decomposes autocatalytically on Fe seed layers (EBID) and on certain electron beam-activated surfaces, yielding high purity, polycrystalline Fe nanostructures. In this contribution, we investigate the growth of structures from Co(CO)_3_NO and compare it to results obtained from Fe(CO)_5_. Co(CO)_3_NO exhibits autocatalytic growth on Co-containing seed layers prepared by EBID using the same precursor. The growth yields granular, oxygen-, carbon- and nitrogen-containing deposits. In contrast to Fe(CO)_5_ no decomposition on electron beam-activated surfaces is observed. In addition, we show that the autocatalytic growth of nanostructures from Co(CO)_3_NO can also be initiated by an Fe seed layer, which presents a novel approach to the fabrication of layered nanostructures.

## Introduction

The fabrication of nanostructures by using focused electron-beam induced processing (FEBIP) techniques, especially electron-beam induced deposition (EBID), has progressed considerably over the last decade [1–5]. In EBID, suitable precursor molecules are dosed onto a surface and then decomposed by the focused electron beam of a scanning electron microscope (SEM) or a transmission electron microscope (TEM). The volatile precursor fragments are pumped off by the vacuum system, while the non-volatile dissociation products remain on the surface as a deposit. Some materials can be deposited with high purity, e.g., iron from iron pentacarbonyl, Fe(CO)_5_ [6–9], cobalt from dicobalt octacarbonyl, Co_2_(CO)_8_ [10–11], or Au from Au(CO)Cl [12]. In addition, EBID offers the advantage of very small obtainable structure sizes [13], the possibility of 3D fabrication, e.g., pillars, and rapid prototyping capabilities [14].

A related FEBIP approach is electron-beam induced surface activation (EBISA) [7]. In EBISA, a suitable substrate, e.g., SiO*_x_* [7,15–18], TiO_2_ [19], or a thin porphyrin film on Ag(111) [8], is irradiated by the focused electron beam in the absence of a precursor, under high vacuum [15] or ultrahigh vacuum (UHV) conditions [7–8,16–19], resulting in a patterned, chemically activated surface. In a second step, a precursor is introduced into the system and decomposes selectively at the irradiated, i.e., activated, areas. Activated in this context means that the corresponding areas are catalytically active towards the decomposition of certain precursor molecules; thereby, an initial deposit (primary structure) can be selectively formed at the pre-irradiated region. The initial deposit might then autocatalytically grow (AG) upon further exposure to the precursor molecules, which allows to produce nanostructures of desired size (secondary growth). Such autocatalytic growth was also observed for primary structures produced by EBID [7]. Reports indicate that the fabrication of primary structures is more effective for EBID compared to EBISA. This might be due to the differences in precursor decomposition for EBID (i.e., in the presence of impinging electrons) and at the pre-activated sites for EBISA [8].

**Figure 1:**
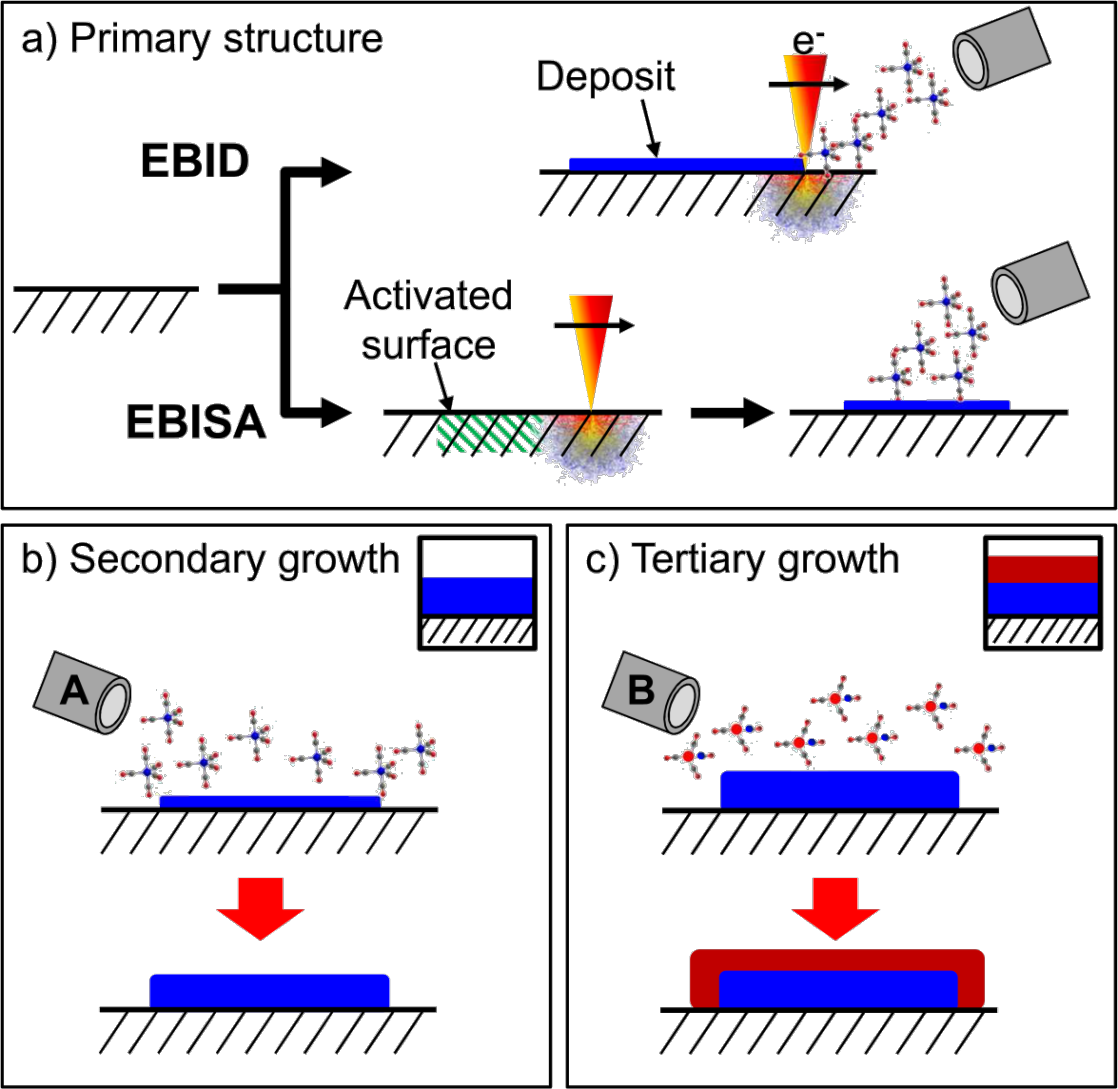
Fabrication and secondary/tertiary growth of nanostructures. The deposits can be fabricated by electron irradiation of a surface in the presence of a precursor (EBID) to form a thin primary deposit (a, top). In the absence of a precursor (a, bottom), some surfaces can undergo selective electron-beam induced surface activation (EBISA), also yielding a primary deposit upon post-exposure to the precursor. If the primary deposits are exposed to the precursor in a successive step, autocatalytic decomposition can lead to further secondary autocatalytic growth of the deposit (b). In the case that a second, different precursor is supplied, another autocatalytic growth process (tertiary growth) can occur, leading to the formation of a layered nanostructure (c). The icons in b) and c) will be used to indicate the respective process throughout this publication.

The EBID and EBISA processes as well as the autocatalytic growth are shown schematically in Figure 1. In addition, the figure introduces a third processing step (c) denoted as “tertiary growth”, in which the structure resulting from a secondary growth process is used as a seed layer for the deposition of another layer of different material by using a different precursor. While this process can be interpreted as a “second secondary growth step”, we will use the term “tertiary growth” throughout this publication to avoid confusion and highlight the sequential nature of the processes.

Depending on the substrate and the precursor, different activation mechanisms have been proposed [7–8,15,18–19]. To be suitable for EBISA, the precursor is required to be susceptible to decomposition only at activated sites. Furthermore, it has to exhibit autocatalytic decomposition in order to facilitate the subsequent growth (also denoted as secondary growth) on top of the primary structure.

Most of the previous EBISA studies as well as some EBID studies used Fe(CO)_5_ as precursor, which yields practically pure, (poly-)crystalline Fe on different substrates [7–8,16–19]. In addition, Co_2_(CO)_8_ was also identified as a suitable precursor for EBISA in experimental work on silica surfaces in a high-vacuum environment [15]. Since other precursors may show a similar behavior, we investigated one relevant candidate concerning autocatalytic growth, namely cobalt tricarbonyl nitrosyl, Co(CO)_3_NO, in more detail. This precursor is more stable and easier to handle than the related Co_2_(CO)_8_. Cobalt tricarbonyl nitrosyl was studied before concerning its ionization properties in the gas phase [20–21], the electron induced decomposition under surface science conditions in UHV [22], and the fabrication and characterization of EBID nanostructures under high vacuum conditions [23–25].

In the gas phase, the decomposition proceeds through direct ionization or dissociative electron attachment depending on the kinetic energy of the involved electrons. Dissociative electron attachment is mainly observed for low-energy secondary electrons (<10 eV) and yields incompletely decomposed fragments, mostly [Co(CO)_2_NO]^−^. Direct ionization occurs for *E* > 10 eV and results in smaller fragments like Co^+^ or [CoCO]^+^ [20–21]. It was suggested that the direct ionization route leads to the deposition of incompletely dissociated precursor molecules, which in turn influences the content of non-metallic contaminants in the deposit [21].

Based on the irradiation of cold (105 K) Co(CO)_3_NO films of about 2.5 nm thickness on amorphous carbon and Au substrates with 500 eV electrons under UHV conditions, the following decomposition mechanism was proposed [22]: At a low electron dose (<5 × 10^16^ *e*^−^/cm^2^), one or two CO molecules are released and the NO ligand decomposes, yielding an adsorbed (CO)*_x_*OCoN species. Upon further electron irradiation at low temperatures, decomposition of CO ligands is observed, yielding carbon-rich (CoO*_y_*N)C_ads_. If instead the initially produced (CO)*_x_*OCoN species is annealed above 244 K, the thermally unstable CO ligands desorb without decomposition, yielding carbon free CoO*_y_*N [22].

At room temperature, EBID using Co(CO)_3_NO in a standard high-vacuum SEM setup yields deposits consisting of about 40–50 atom % Co, 25–35 atom % O, 10–15 atom % N and 10–15 atom % C as determined by energy-dispersive X-ray spectroscopy (EDX) [23–25]. The composition is almost independent of the applied beam current and energy, apart from a slight increase in oxygen content for increasing beam power [23]. The deposition yield decreases for higher electron energy, and increases strongly above 403 K substrate temperature [23]. A more detailed study addressed the temperature dependence for various precursors. For Co(CO)_3_NO and Co_2_(CO)_8_ three distinct regimes were proposed: (1) EBID only, (2) seeded growth, i.e., enhancement of deposition rate and autocatalytic growth, and (3) spontaneous decomposition and film growth, i.e., chemical vapor deposition (CVD) [24]. For Co(CO)_3_NO, EBID was found up to about 393 K, followed by seeded growth up to about 403 K and spontaneous decomposition at higher temperatures. In the EBID regime, increasing the temperature from 293 to 323 K lowered the carbon content by a factor of three. In addition, the oxygen content decreased and the nitrogen content increased with temperature, while the cobalt content remained almost constant. In the seeded and spontaneous growth regimes, the composition remained constant at about 50 atom % Co, 20–25 atom % O and N, and a few atom % C.

In the present study, the autocatalytic growth of nanostructures by using Co(CO)_3_NO at room temperature is investigated and compared to that using Fe(CO)_5_. EBID structures prepared from Co(CO)_3_NO in a UHV environment are exposed to additional Co(CO)_3_NO to induce autocatalytic growth; the resulting deposits are characterized by SEM and scanning transmission X-ray microscopy (STXM). STXM allows for the non-destructive quantitative spectromicroscopic characterization of the individual layers with nanoscale resolution and high contrast due to the possibility of resonant imaging [26]. The EBID deposits are compared to deposits produced by EBISA with Co(CO)_3_NO, and to deposits prepared by autocatalytic growth of Co(CO)_3_NO on iron seed layers, which were prepared beforehand by EBID with Fe(CO)_5_. The latter process opens up a novel approach for the localized fabrication of arbitrarily shaped bilayer and even multilayer nanostructures.

## Results and Discussion

### EBID plus autocatalytic growth

**Figure 2:**
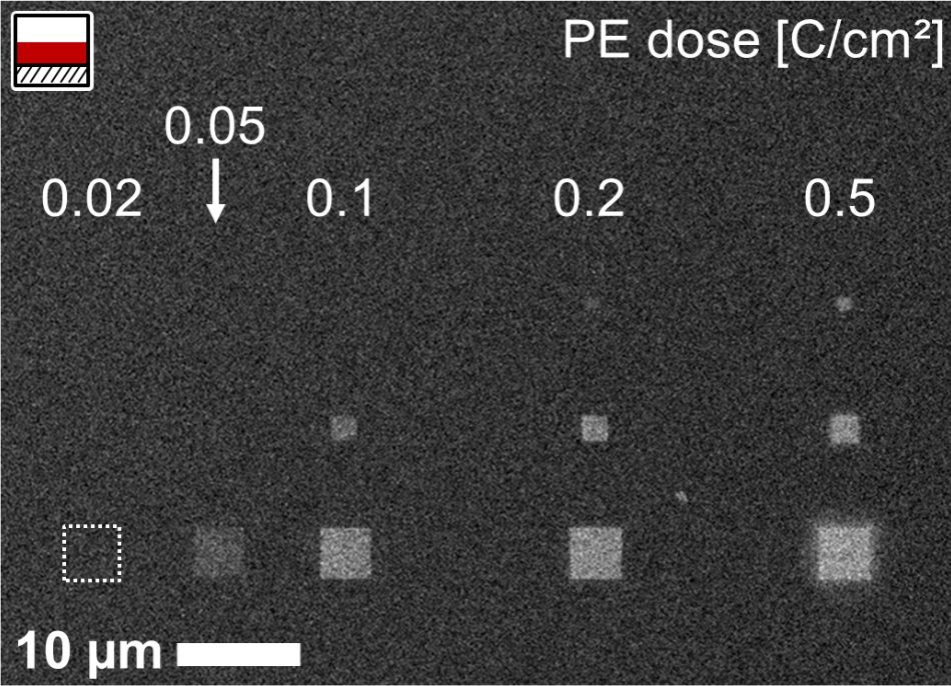
SEM micrograph of square EBID structures of different sizes and primary electron doses (as indicated), prepared on a 100 nm Si_3_N_4_ membrane using Co(CO)_3_NO. The structures were prepared in one experimental run, from left to right, top to bottom. After the EBID step, the precursor was further supplied to allow for autocatalytic growth. The total growth time increases from 198 min (bottom right structure) to 230 min (top left structure). The minimum electron dose for detectable deposition is about 0.05 C/cm^2^, while a dose of 0.5 C/cm^2^ marks the start of proximity effect-induced loss of structure definition (fringe surrounding bottom right structure).

EBID structures were deposited from Co(CO)_3_NO on native SiO*_x_* on Si(100) and 100 nm Si_3_N_4_ membranes, and on commercially available, thermal 300 nm SiO_2_ on Si(100). The beam energy was 15 keV at a beam current of 400 pA; the step size was 6.2 nm. Figure 2 displays SEM images of square structures (1 × 1, 2 × 2 and 4 × 4 µm^2^) on the native oxide on Si_3_N_4_, which were irradiated with primary electron (PE) doses ranging from 0.02 to 0.5 C/cm^2^, at a precursor pressure of approx. 9 × 10^−6^ mbar. The irradiation of each individual structure was performed by successively sweeping the same area 10 times (rather than in a single sweep). This procedure enhances the uniformity of the fabricated structures, which otherwise shows a pronounced asymmetry due to proximity effects (see Figure S1 in Supporting Information File 1 for details). In Figure 2 the electron dose increases from left to right, and the size from top to bottom. The structures were written sequentially, left-to-right and row-by-row in one experimental run: The EBID process lasted 32 min, and thereafter, the precursor pressure was maintained to induce autocatalytic growth. The deposition process, including the EBID step, lasted 230 min, which corresponds to an accumulated precursor dose of about 9.3 × 10^4^ Langmuir (1 L = 10^−6^ Torr·s ≈ 1.33 × 10^−6^ mbar·s). Since EBID of the individual deposits was performed sequentially, the respective times for autocatalytic growth after EBID varied from 230 min for the low dose structures on the top left corner (exposed first) to 198 min for the high dose structures on the bottom right (exposed last). The structures appear brighter for higher doses: While below 0.05 C/cm^2^ no structure can be unequivocally identified, a dose of 0.5 C/cm^2^ marks the start of observable proximity effects in the form of fringes around the structures. Closer inspection of the structures shows that, despite the same electron dose was applied per surface area, larger squares are brighter and more defined compared to the smaller ones, which points to a deposition that is influenced by proximity effects [2].

**Figure 3:**
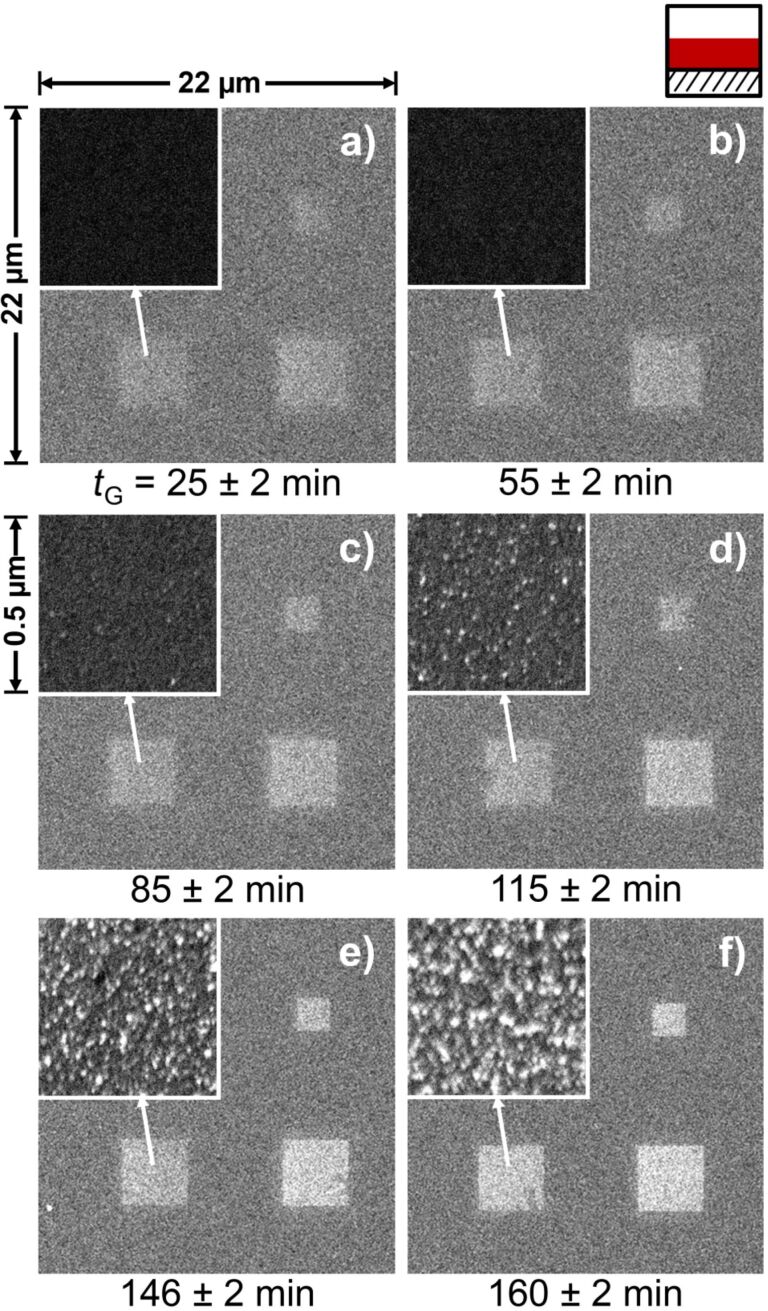
Nanostructures prepared by EBID plus autocatalytic growth using Co(CO)_3_NO on Si_3_N_4_ with different growth times, as indicated below the images. The electron dose during EBID was 0.2 C/cm^2^ for the structures in the right column of every image; the dose of the lower left square was 0.1 C/cm^2^. The insets show higher magnification images of the respective structures directly below. With increasing growth time the images appear brighter, and the granular nature of the deposits is more pronounced.

In addition to the dose dependence, the growth time-dependent appearance of the structures was investigated. Figure 3 compares SEM images of square deposits fabricated by EBID and autocatalytic growth, using Co(CO)_3_NO as precursor. The growth time, *t*_G_, was varied from 25 to 160 min. In each of the six images (a–f), the two squares in the right column were irradiated with a primary electron dose of 0.2 C/cm^2^, and the square in the left column with 0.1 C/cm^2^. The inset in each case shows the morphology of the respective structures at 44× higher magnification.

The data in Figure 3 shows a direct correlation between the brightness of the structures in SEM and the applied growth time, indicating an increased material deposition. The brighter appearance of the deposited structures at larger growth times is attributed to an enhanced yield of backscattered electrons (BSE) and thereby induced secondary electrons. Indeed, Monte-Carlo simulations using the software CASINO (v. 2.42) [27] show an increase in the BSE emission coefficient by more than 20% for a 5 nm layer of Co_0.51_O_0.24_N_0.14_C_0.11_ (composition reported by Gazzadi et al. [23]) and by over 40% for a 5 nm layer of pure Co compared to the bare 100 nm Si_3_N_4_ membrane. The high-magnification insets in Figure 3 reveal the formation of a strongly corrugated, granular deposit, which can be interpreted as the growth of small clusters of material. Both the increase in the brightness of the deposits and the cluster growth mode are in line with the autocatalytic growth of EBID deposits upon dosage of additional Co(CO)_3_NO.

**Figure 4:**
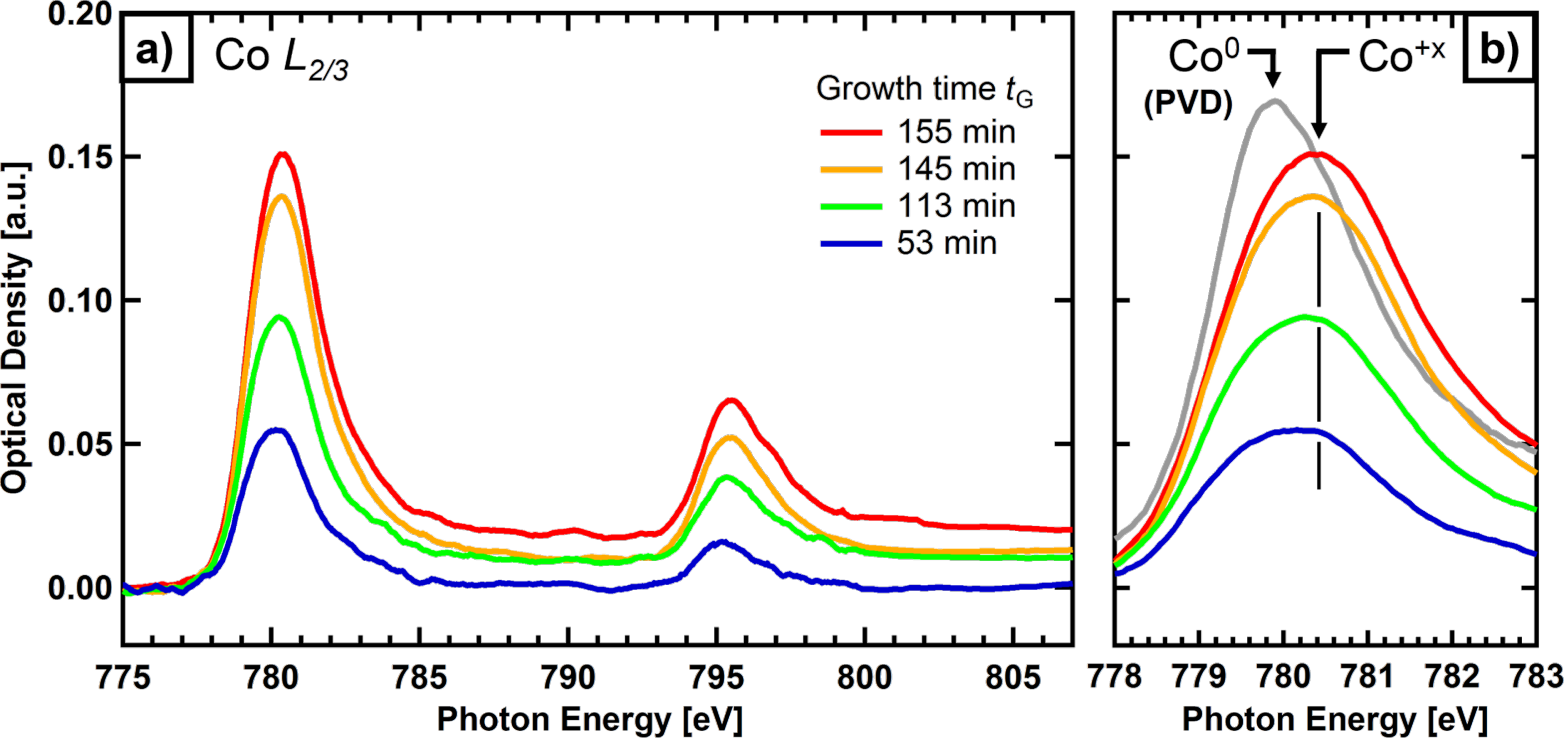
(a) Co L-edge X-ray absorption spectra of deposits prepared on a Si_3_N_4_ membrane by EBID (0.2 C/cm^2^) plus autocatalytic growth using Co(CO)_3_NO, with different growth times (indicated by colors); the spectra were vertically shifted to a common pre-edge baseline. (b) L_3_ edge of the deposits at an enlarged photon energy scale, along with the spectrum of a Co layer produced by PVD as reference (grey).

The samples were further characterized at the PolLux soft X-ray STXM beamline [28] at the Swiss Light Source using a zone plate with a nominal resolution of 30 nm and a photomultiplier tube (PMT) behind the specimen for X-ray detection in transmission mode. The STXM was operated under high-vacuum conditions (low 10^−6^ mbar range) to reduce contamination from the decomposition of residual gases. In Figure 4, Co L-edge spectra of deposits prepared by EBID (0.2 C/cm^2^) and autocatalytic growth with Co(CO)_3_NO are presented for different growth times. The left panel (a) shows an overview of the Co L_2/3_ region; the right panel (b) an enlargement of the L_3_ region along with the spectrum of a layer of pure cobalt (grey) that was produced by physical vapor deposition (PVD).

The comparison of the Co peak positions (maxima) of the metallic cobalt film prepared by PVD (779.9 eV, Co^0^) and the structures prepared by EBID plus autocatalytic growth (780.4 eV) reveals a chemical shift of approx. 0.5 eV, which is indicative of cobalt in an oxidized state ([29] and references [8] and [9] therein). This suggests a composition in the form of CoO*_x_*N*_y_*(C*_z_*), in line with previous reports for comparable EBID structures by Gazzadi et al. [23,25] and Mulders et al. [24].

The observed intensities in Figure 4 are a direct measure of the layer thickness of the deposits on the Si_3_N_4_ membrane. In transmission X-ray microscopy or NEXAFS spectroscopy in transmission mode, the absorbance (or optical density, OD) is derived from:

[1]
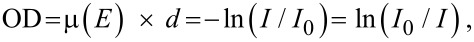


with *I*_0_ and *I* being the incident and the transmitted intensities, respectively, *d* represents the sample thickness and µ*(E)* the photon energy dependent linear absorption coefficient. The peak shape and the energy of the resonant Co L_3_ transition are similar for all deposits, which supports a common attenuation coefficient for this energy. The spectral intensities in Figure 4 also indicate that the layer thickness increases with growth time. As the analysis is based on the Co L_3_ signal, this increase is unequivocally related to the growth of a layer of Co-containing material by autocatalytic decomposition of Co(CO)_3_NO.

In order to quantify the absorption of the deposits, images were recorded at the resonant transition peak at 780.4 eV. The resonant transition yields the strongest element-specific absorption and thus maximizes the image contrast for ease of evaluation. The optical density of the structures was calculated by averaging the signal over the area of the respective structure (in the STXM micrograph) and referencing the signal to the background, i.e., the signal of the pristine membrane near the deposit. The granular structure of the deposits, which was observed in SEM (cf. Figure 3), could not be observed in STXM due to the limited resolution of the applied zone plate of approx. 30 nm.

As the exact chemical composition of the deposit is not known, the linear absorption coefficient of the deposited material, µ_deposit_, is also unknown. As an approximation, we use the value of pure Co, µ_Co_, instead and denote the derived thickness value as apparent cobalt thickness, *d*_A_, which is calculated by using Equation 1. Since the oxygen, nitrogen and carbon contributions are small compared to resonant Co L_3_ signals, *d*_A_ is considered a meaningful value, reflecting the nominal thickness of a pure Co layer. The real thickness of the deposit is certainly underestimated, but since the composition of the different EBID deposits is very likely the same, a comparison of the deposits obtained with different growth times is possible (see section Experimental for more details).

**Figure 5:**
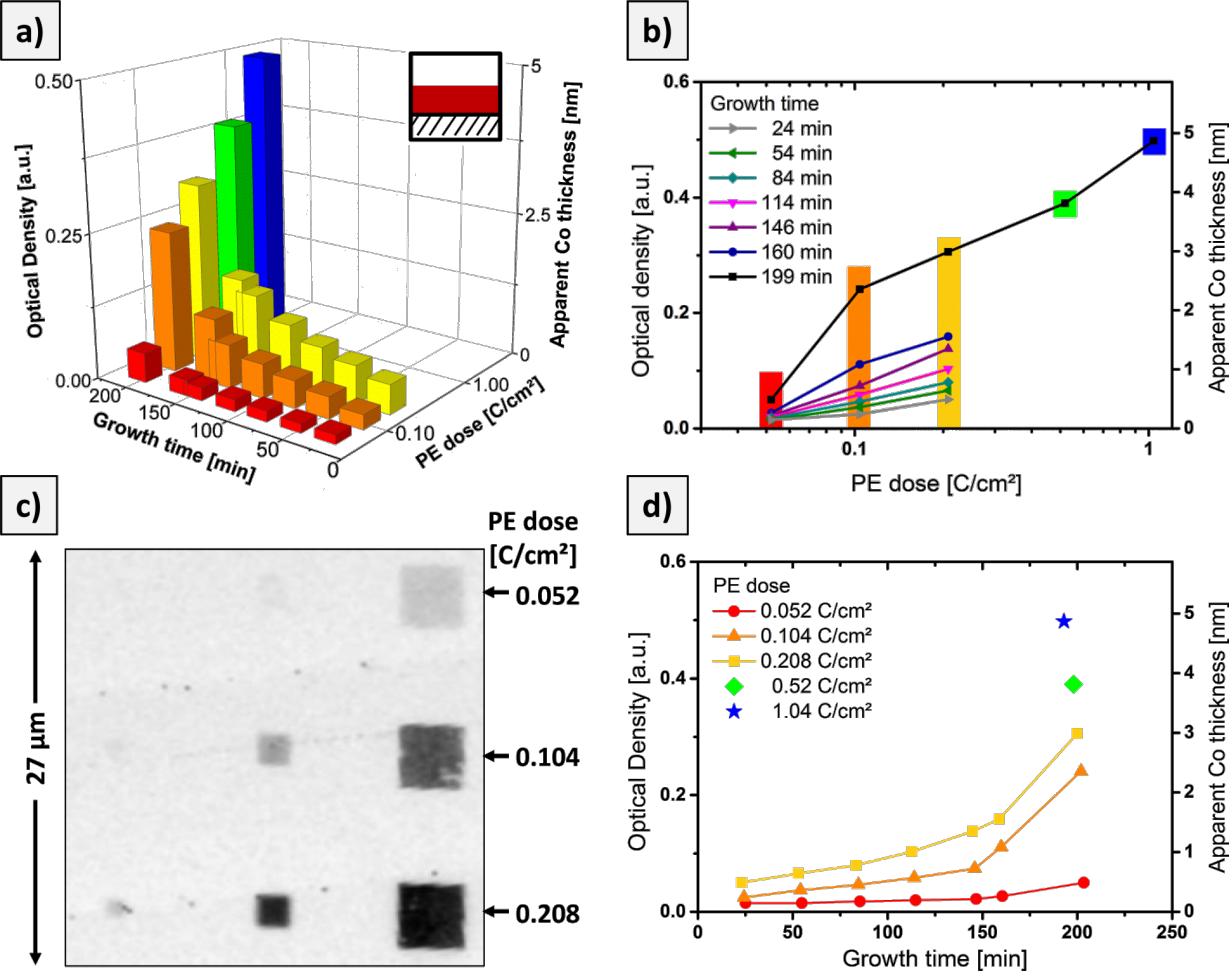
Evaluation of the X-ray absorption data for the growth of Co-containing deposits by EBID plus autocatalytic growth upon Co(CO)_3_NO dosage. a) Optical density (left vertical axis) and apparent Co thickness (right vertical axis) of 4 × 4 µm^2^ squares vs growth time and PE dose during EBID. b) and d) detailed graphs of the observed growth behavior; the color code identifies the respective data set. c) STXM micrograph (transmitted intensity) of a set of deposits prepared with different PE doses, but the same growth time of about 160 min obtained at a photon energy of 780.4 eV. See text for the definition of the apparent Co thickness.

Figure 5 summarizes the thickness analysis for several deposits produced by EBID plus autocatalytic growth as a function of the primary electron dose during EBID and the growth time, *t*_G_, during which Co(CO)_3_NO was continually supplied. The 3D plot in Figure 5a shows the optical density (left vertical axis) and apparent Co layer thickness (right vertical axis) vs growth time and primary electron dose (log scale), and Figure 5b and Figure 5d show the detailed plots against the latter two parameters. The STXM micrograph (transmitted intensity) in Figure 5c displays a set of square deposits fabricated with the indicated primary electron doses and a growth time of about 160 min. The data in Figure 5 are in line with our previous observations: The optical density increases with both electron dose and autocatalytic growth time. The dependence on the primary electron dose in Figure 5b can be linearly extrapolated to zero, in order to obtain the minimum dose required for direct EBID, yielding a value of 0.03 ± 0.01 C/cm^2^ or 1.8 ± 0.6 × 10^3^ electrons per nm^2^. The dependency of the optical density on the growth time in Figure 5d (for a given primary electron dose) initially exhibits an almost linear behavior, but for *t*_G_ > 150 min a strong nonlinear increase is apparent. The morphology of the deposits in Figure 3 indicates that the growth proceeds in a granular fashion and not by homogeneous layer-by-layer growth. For this complex growth process, during which the number of available sites, the (local) precursor concentration or both may vary, a nonlinear behavior is to be expected.

### EBISA plus autocatalytic growth

The susceptibility to decomposition by an electron beam-activated surface or via an autocatalytic process is a prerequisite for the successful application of a precursor for EBISA-based fabrication of nanostructures. In order to study the suitability of Co(CO)_3_NO, a number of test patterns were irradiated on different surfaces under UHV conditions and subsequently exposed to Co(CO)_3_NO. The investigated surfaces were SiO*_x_* layers on Si(100) and Si_3_N_4_, both of which are suitable substrates for EBISA using Fe(CO)_5_ [7,16]. On these surfaces, electron stimulated desorption of oxygen and the thereby created oxygen vacancies were identified as the active sites for the initial decomposition of Fe(CO)_5_ [7,18]. However, the corresponding experiments with Co(CO)_3_NO as a precursor in EBISA were not successful, i.e., deposition of material on the activated surfaces was not observed (data not shown). We thus have to conclude that Co(CO)_3_NO is not suitable as precursor for the fabrication of nanostructures by using EBISA on silicon oxide surfaces.

An alternative approach could be to use different substrates for EBISA: It was shown recently by our group that it is possible to activate thin layers of large organic molecules (2*H*-tetraphenyl porphyrin) on metal single crystals for Fe(CO)_5_ decomposition [8]. The proposed activation mechanism involves the electron-beam induced formation of reactive organic moieties, which might be reactive also towards the initial decomposition of Co(CO)_3_NO. Such investigations are, however, out of the scope of the present study.

### Autocatalytic growth on iron seed layers

In addition to the experiments described before, the fabrication of layered nanostructures by EBID using both Fe(CO)_5_ and Co(CO)_3_NO was explored. In the course of these experiments, we observed that Co(CO)_3_NO does not only decompose autocatalytically on Co-containing deposits (such as the EBID deposits discussed before), but also on high purity Fe nanostructures. The latter can be prepared from Fe(CO)_5_ by EBID or EBISA, plus successive autocatalytic growth.

The iron structures are typically composed of very pure (>90–95 atom %) cubic crystallites, as a result of the autocatalytic growth process [7–8,16–19]. The morphology ranges from scattered clusters for low electron doses and shorter growth times, to fused, polycrystalline patches of cubic crystallites for high electron doses and long growth times [7–8,16–19]. After preparation of the Fe deposits with Fe(CO)_5_, Co(CO)_3_NO was introduced into the chamber for a given growth time that was identical for all Fe seed deposits. Thereafter, the samples were investigated by SEM (not shown) and STXM. For the STXM analysis, images were acquired at the Fe L_3_ and Co L_3_ absorption edges. To determine the thickness of the Co layer, the absorption by the Fe layer underneath has to be considered: Whereas the absorption by Co at the Fe L_3_ edge (708.7 eV) is negligible, the absorption by Fe at the Co L_3_ edge (780.4 eV) is considerable. By comparison of the optical density (OD) of pure iron deposits at both energies, a contribution of (25 ± 5)% of the Fe intensity at the Fe *L_3_* edge is determined for the Co L_3_ edge, i.e.,


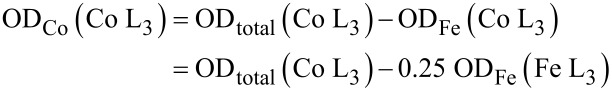


This correction was taken into account to determine the apparent thickness of the Co contribution in the CoO*_x_*N*_y_*C*_z_*/Fe bilayer.

**Figure 6:**
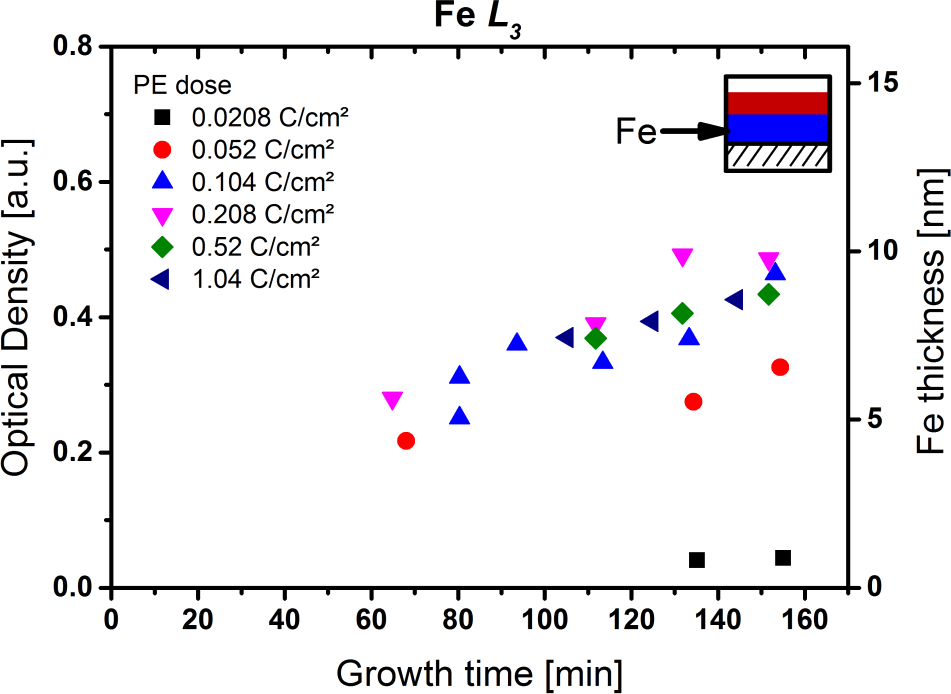
Optical density at the Fe L_3_ edge at 708.7 eV (left vertical axis) and average thickness of the iron layer (right vertical axis) of various CoO*_x_*N*_y_*C*_z_*/Fe nanostructures versus autocatalytic growth time for Fe(CO)_5_. The different symbols indicate different primary electron (PE) doses. Above 0.05 C/cm^2^, the thickness/optical density increases linearly with the growth time at a rate of 0.5 ± 0.1 Å/min irrespective of the applied PE dose. Lower PE doses (red, black) exhibit reduced thickness.

As the first step, the autocatalytic growth of the iron structures was investigated. Figure 6 shows an overall linear increase of the optical density (left vertical axis) and the average thickness (right vertical axis) with autocatalytic growth time for the Fe L_3_ edge. For electron doses above 0.05 C/cm^2^, the data for different PE doses are very similar. This indicates that the Fe layer thickness is mainly determined by the autocatalytic growth time, with an autocatalytic growth rate of 0.5 ± 0.1 Å per minute (approx. 1.3 × 10^−3^ Å/Langmuir). The observation that for electron doses of 0.05 C/cm^2^ and below only reduced thicknesses are obtained indicates that the threshold for creating a homogenously reactive initial deposit by EBID is not yet reached. Thus, only for electron doses exceeding 0.05 C/cm^2^, the number of catalytically active sites per area approaches a saturation value. This induction period is followed by a constant rate of autocatalytic precursor decomposition, which results in constant height growth. It is likely that the deposit formed in the induction period is a closed layer of iron on the surface. These results confirm that continued deposition of Fe is possible on the initial layer prepared by EBID with comparatively low electron doses and thus short fabrication times, as was observed before [7–8,16–19].

In a next step, the high purity Fe structures are exposed to Co(CO)_3_NO. This results in the deposition of a layer of material containing cobalt on top of the Fe structure in a tertiary growth process. The deposition is selective, i.e., the Co-containing layer is only observed on the Fe structures while the pristine membrane remains uncovered. The composition of the Co-containing layers is most likely again CoO*_x_*N*_y_*C*_z_*, which is supported by the shift of the Co L_3_ peak to higher energy, and by Auger electron spectroscopy of comparable structures on SiO*_x_*/Si(100) (not shown); note that severe charging prevents Auger electron spectroscopy on the Si_3_N_4_ membrane samples.

**Figure 7:**
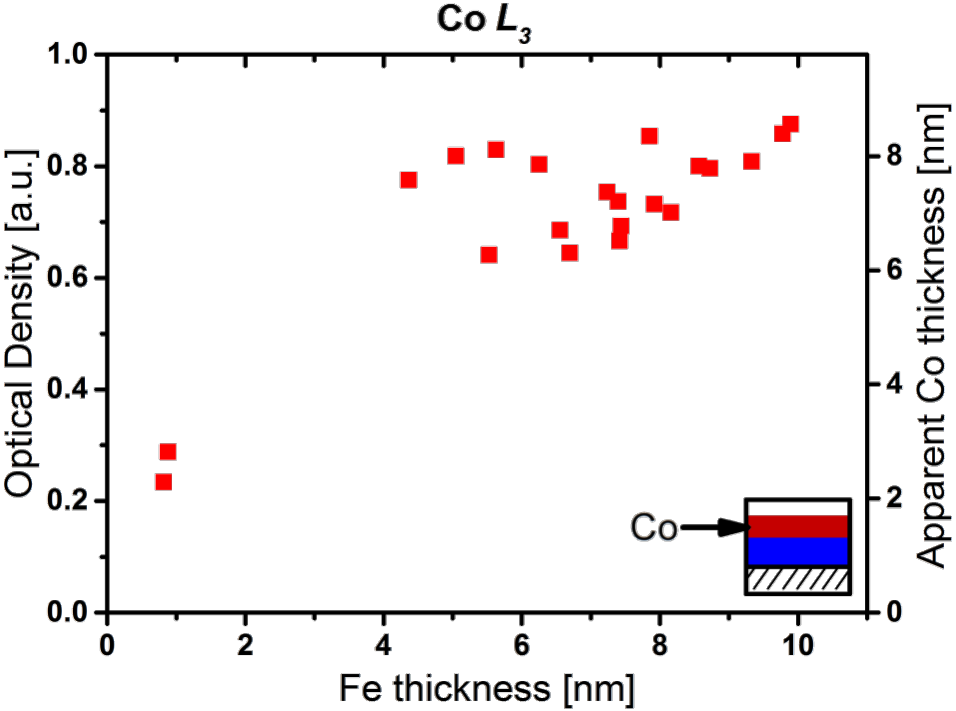
Optical density at the Co L_3_ edge and apparent cobalt thickness of CoO*_x_*N*_y_*C*_z_* layers grown autocatalytically from Co(CO)_3_NO on Fe layers plotted against the Fe layer thickness. The Fe layers were prepared by EBID and autocatalytic growth by using Fe(CO)_5_. In a second step, Co(CO)_3_NO was dosed for 210 min to produce the CoO*_x_*N*_y_*C*_z_* layers via (auto-)catalytic decomposition. The optical density and thickness are corrected to account for the absorption of the Fe deposit underneath (see text). The OD of the CoO*_x_*N*_y_*C*_z_* layer is almost independent of the Fe layer thickness for Fe layers thicker than 4 nm.

Figure 7 shows the optical density (left vertical axis) at the Co L_3_ edge and average apparent Co thickness *d*_A_ (right vertical axis) of CoO*_x_*N*_y_*C*_z_* layers grown on iron seed layers of increasing thickness (corresponding to the layers in Figure 6). In all cases the same total growth time (210 min) using Co(CO)_3_NO was applied. On top of an Fe layer thicker than 4 nm, a comparable optical density of 0.76 ± 0.08 is observed for the autocatalytically grown CoO*_x_*N*_y_*C*_z_* layers, independent of the thickness of the initial Fe layers. This can be interpreted as being due to very similar starting conditions for the tertiary growth process on all investigated Fe layers. It is reasonable to assume that the Fe deposits are closed layers of Fe, which provide identical densities of active sites for the initial decomposition of Co(CO)_3_NO. The nature of the active site cannot be deduced from the available data, but we assume that upon adsorption of the Co(CO)_3_NO precursor on the clean Fe film the immediate dissociation of the precursor takes place. In order to gain more insight into the underlying reaction(s), chemically more sensitive methods like XPS and IR spectroscopy may be helpful.

The apparent cobalt thickness observed on the thick Fe seed layers is 7.4 ± 0.8 nm; the average growth rate is 0.35 ± 0.05 Å/min. It is likely, however, that the growth on Fe seeds exhibits non-linear behavior, as was observed above for the autocatalytic growth on the cobalt seed layer (cf. Figure 5).

Comparing the growth behavior of Co(CO)_3_NO and Fe(CO)_5_*,* the presented data (Figure 6 vs Figure 5d) suggest two very different growth modes for the different precursors under otherwise identical reaction conditions. On the one hand, the autocatalytic decomposition of Fe(CO)_5_ proceeds at a constant rate and produces high purity Fe deposits, which (above a threshold) are almost independent of the applied PE dose. On the other hand, the autocatalytic decomposition of Co(CO)_3_NO exhibits pronounced non-linear, possibly even exponential behavior and is strongly influenced by the applied primary electron dose during the initial EBID step. In contrast to Fe(CO)_5_, the decomposition yields an oxygen-, nitrogen- and carbon-rich CoO*_x_*N*_y_*C*_z_* deposit instead of pure cobalt. Besides the involved chemistry, which is likely to be quite different, yet difficult to study with the available techniques, the deposits structure seems to have a strong influence on the growth behavior. As was already reported before [7–8,16–19], iron structures fabricated by EBID/EBISA plus autocatalytic growth are composed of cubic crystallites of α-Fe(bcc). These crystallites are quite regular, and their vacuum interfaces consist mainly of low index {100} faces of these cubes. A representative structure is presented in Figure S2 of Supporting Information File 1. In the case of Co(CO)_3_NO, not very well-defined granular structures are observed after the autocatalytic growth step, indicating a rather amorphous and defect rich deposit with a high surface area. Such structures are likely to show different, possibly increased reactivity compared to ordered, flat surfaces. Indeed, the observed decomposition of Co(CO)_3_NO on the nominally flat Fe seed layers is less pronounced than on the Co seed layer produced with very high primary electron doses.

## Conclusion

We have investigated the electron-beam induced decomposition of Fe(CO)_5_ and Co(CO)_3_NO and the subsequent secondary growth via selective autocatalytic decomposition upon further precursor dosage. The two precursors show very different growth characteristics under the applied reaction conditions. Structure fabrication by using Co(CO)_3_NO is strongly affected by the applied electron dose in the EBID step and subsequent autocatalytic growth time. The influence of the electron dose follows a logarithmic trend, while the autocatalytic thickness growth shows non-linear, possibly exponential behavior with growth time. This is explained by the observed granular morphology of the deposits and the associated high surface area, defect rich, and reactive deposit–vacuum interface. The analysis of the chemical composition of the structures prepared from Co(CO)_3_NO points to an oxygen-, nitrogen-, and carbon-rich CoO*_x_*N*_y_*C*_z_* composite material, with the Co L_3_ peak shifted towards an oxidic state. Fe(CO)_5_ exhibits a constant growth rate of 0.5 ± 0.1 Å/min, which above a threshold of 0.05 C/cm^2^ is almost independent of the electron dose applied during the initial EBID step. The deposits prepared by EBID/EBISA and autocatalytic growth from Fe(CO)_5_ are composed of polycrystalline, high purity Fe (typically more than 90–95 atom %). While the electron irradiation defines the shape of the deposit, the thickness of the prepared structures is governed mainly by the autocatalytic growth process. The practical separation of shape definition and deposit formation has some advantages, most obviously the reduction of proximity effects due to lower required electron dose as compared to the EBID-only process.

In order to fully understand the underlying mechanism of the autocatalytic decomposition especially of Co(CO)_3_NO, it is necessary to conduct further studies on model systems using complementary surface science techniques, e.g., X-ray photoelectron spectroscopy (XPS) or infrared absorption spectroscopy (IR/IRAS), and expand the work that has been done on the electron-beam induced decomposition to include the autocatalytic growth.

Our study also shows that the EBISA approach does not work with Co(CO)_3_NO as a precursor: While Fe(CO)_5_ decomposes on activated, i.e., electron pre-irradiated, areas of SiO*_x_* surfaces and forms a deposit, this behavior was not observed for Co(CO)_3_NO. Interestingly, Co(CO)_3_NO decomposes autocatalytically on Fe seed layers, which opens up a number of fabrication possibilities. As the fabrication of Fe structures by EBISA plus autocatalytic growth has been shown to be a successful approach not only on SiO*_x_* surfaces, but also on TiO_2_ [19] and on substrates pre-covered with organic layers [8], such Fe layers could be generally considered as seeding layers for precursors that are not susceptible to decomposition by the electron-beam activated surface. Furthermore, the fabrication of layered nanostructures without the necessity for multiple electron beam exposure steps was demonstrated. It is likely that such a seeding concept also works for other precursor combinations. The known autocatalytic growth of high purity Co deposits from Co_2_(CO)_8_ [15] makes that precursor an obvious candidate for the fabrication of layered Co/Fe nanostructures with arbitrary shapes.

Thus, the presented results considerably expand the possibilities of FEBIP-based nanofabrication techniques. We also show that the potential for (auto-)catalytic decomposition of typical EBID precursors needs to be studied in detail. This approach is necessary to gain a deeper understanding of the underlying processes, the consequences of autocatalytic growth for EBID experiments and, subsequently, to develop new or improved methods for the fabrication of FEBIP-based nanostructures.

## Experimental

All structures were fabricated in a commercial UHV system (Multiscanlab, Omicron Nanotechnology, Germany) with a base pressure of *p* < 2 × 10^−10^ mbar. The system consists of a UHV-compatible electron column (Leo Gemini) for scanning electron microscopy (SEM, nominal resolution better than 3 nm), electron beam based lithography (EBL, EBID), local Auger electron spectroscopy (AES) and scanning Auger microscopy (SAM), with a resolution better than 10 nm using a hemispherical electron energy analyzer. All electron exposures for SEM and lithography were performed at a beam energy of 15 keV and a current of 400 pA. The lithographic processes were controlled through a custom-developed software based on LabVIEW 8.6 (National Instruments) and a high-speed DAC PCIe-card (M2i.6021-exp, Spectrum GmbH, Germany). SEM images were acquired with *SmartSEM* (Zeiss) and are shown with minor contrast and brightness adjustments only.

The precursors were purchased from ACROS Organics (iron pentacarbonyl, Fe(CO)_5_) and abcr GmbH & Co. KG (cobalt tricarbonyl nitrosyl, Co(CO)_3_NO). The purity of the precursor gas was analyzed with a quadrupole mass spectrometer in a dedicated gas analysis chamber (base pressure below 2 × 10^−9^ mbar).

The precursor gas was dosed onto the sample surface through a nozzle. Based on simulations using GIS Simulator (version 1.5) [30], the local pressure increase on the sample surface was calculated to be a factor of 30. For a fixed background pressure of 3.0 × 10^−7^ mbar, this corresponds to a local pressure at the surface of about 9 × 10^−6^ mbar [31].

Si_3_N_4_ samples (TEM size holder SI frame, 500 µm × 500 µm membrane size, thickness 100 nm) were supplied by Plano GmbH. Si(100) samples were purchased from the Institute of Electronic Materials Technology, Warsaw, Poland.

STXM experiments were performed at the PolLux beamline at the Swiss Light Source (SLS), Paul Scherrer Institut, Villigen (CH) [28]. The standard STXM setup uses high brilliance synchrotron radiation light that is focused on the sample by a Fresnel zone plate. The sample is raster-scanned with interferometric control through the focal spot, while the transmitted photon intensity is recorded by using a photo multiplier tube. Near-edge X-ray absorption fine structure (NEXAFS) spectra were recorded by consecutive scanning of the investigated area with varying photon energy. The lateral resolution in routine operation for the applied zone plate was 30 to 40 nm. The STXM data were analyzed using aXis2000 (http://unicorn.mcmaster.ca/aXis2000.html).

The value for the absorption coefficient at the resonant transition was determined by fitting the spectrum of a PVD Co layer to a theoretical spectrum (“X-ray Form Factor, Attenuation, and Scattering Tables”; NIST [32–34]), which do not account for resonant transitions. The fit is accomplished by scaling the optical density of the measured spectrum of the Co layer to match the pre-edge and post-edge region of the theoretical spectrum of 1 nm thick Co. The scaled spectrum, which contains the resonant transition, then allows to determine µ(*E*) values. The linear attenuation coefficient for Co at the resonant transition was found to be µ_Co_(779.9 eV) = 0.103 ± 0.02 nm^−1^. For the quantification of the apparent cobalt thickness of the CoO*_x_*N*_y_*C*_z_* deposits, we assume that the absorption coefficient of the resonant peak intensity is comparable for pure Co and CoO*_x_*N*_y_*C*_z_*, i.e., µ_Co_(779.9 eV) ≈ µ_CoO_*_x_*_N_*_y_*_C_*_z_*(780.4 eV).

The absolute thickness of the CoO*_x_*N*_y_*C*_z_* deposits may be three to five times greater than the reported apparent Co thickness according to our estimations. The absorption coefficient µ_Fe_(708.7 eV) = 0.050 ± 0.01 nm^−1^ was also determined using the described fitting procedure.

## Supporting Information

Supporting Information contains additional SEM images of proximity effects during EBID of Co(CO)_3_NO and examples of Fe deposits prepared by EBID/EBISA and autocatalytic growth using Fe(CO)_5_ as a precursor on native oxide on a silicon nitride membrane.

File 1 Additional SEM images.
