# Supplementary material for: Electron-beam induced deposition and autocatalytic decomposition of Co(CO)3NO
Source: Beilstein J Nanotechnol. 2014 Jul 30;5:1175–85. doi: 10.3762/bjnano.5.129 (PMC4143096; doi:10.3762/bjnano.5.129)
Supplement: File 1 — Additional SEM images. [file Beilstein_J_Nanotechnol-05-1175-s001.pdf]

Supporting Information

for

Electron-beam induced deposition and autocatalytic decomposition of $Co(CO)_3NO$

Florian Vollnhals, Martin Drost, Fan Tu, Esther Carrasco, Andreas Späth, Rainer H. Fink, Hans-Peter Steinrück and Hubertus Marbach*

Address: Lehrstuhl für Physikalische Chemie II and Interdisciplinary Center for Molecular Materials (ICMM), Friedrich-Alexander-Universität Erlangen-Nürnberg, Egerlandstr. 3, 91058 Erlangen, Germany

Email: Hubertus Marbach* - hubertus.marbach@fau.de

*Corresponding author

Additional SEM images

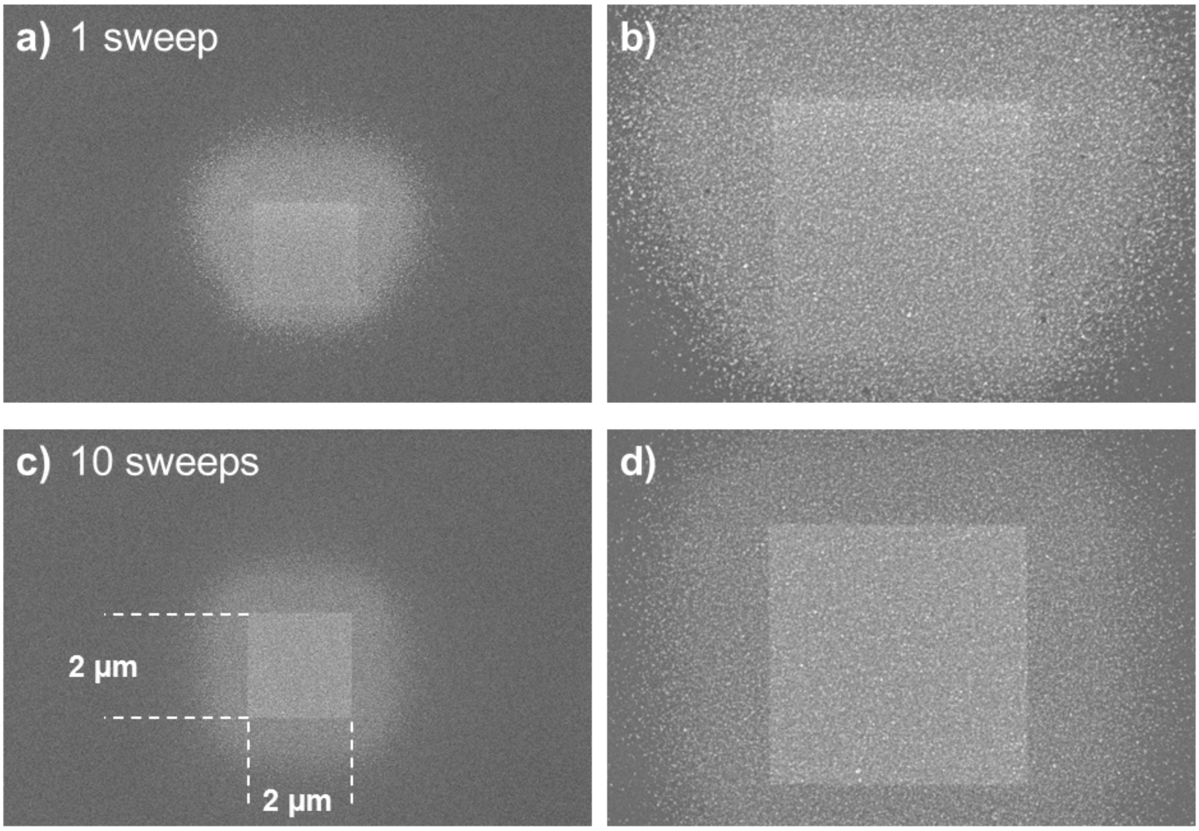

Figure S1: SEM of 2 × 2 µm² deposits prepared by EBID and autocatalytic growth using $Co(CO)_3NO$ on a 100 nm Si_3N_4 membrane (a, c) and corresponding high resolution data (b, d). The total PE dose is 0.55 C/cm², applied in one sweep with a dwell time of 0.5 ms (a) or in ten sweeps with a dwell time of 0.05 ms per sweep (c). In the single top-to-bottom sweep case (a) the top edge appears brighter and is surrounded by a pronounced bright fringe, while the bottom edge fades into the background. This behavior is attributed to proximity effects caused by electron scattering at already deposited material. In the case of multisweep irradiations, the deposit presents a more uniform distribution of material. The lower dwell time per sweep hinders the formation of a pronounced starting edge deposit, reducing the proximity effects. The high resolution images b) and d) also confirm the granular nature of the autocatalytically grown $CoO_xN_yC_z$ deposit. The less pronounced granularity observed in d) is attributed to the lower growth time (a/b: 260 min, c/d: 220 min).

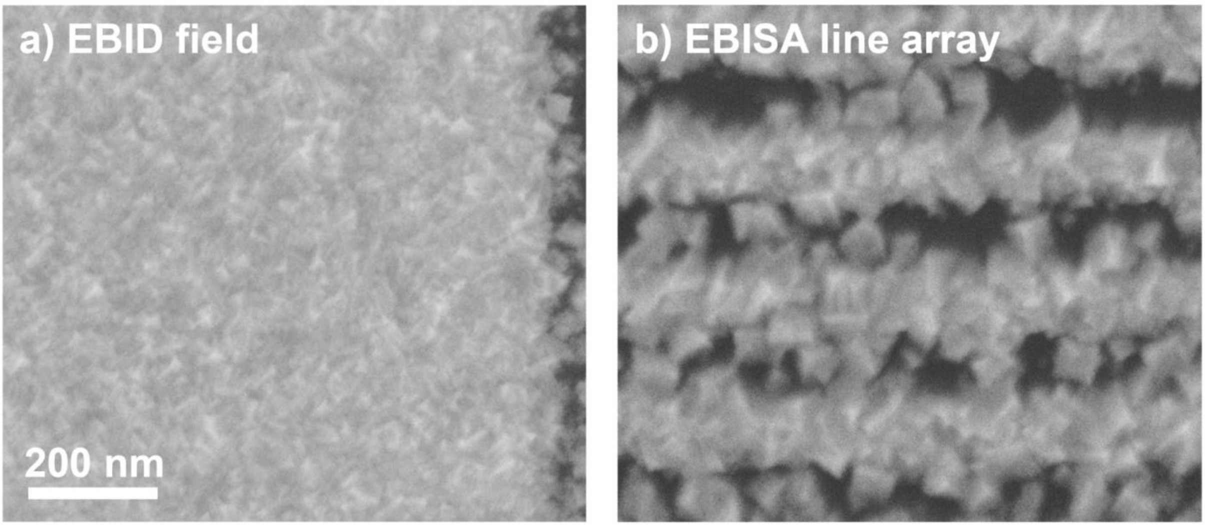

Figure S2: Scanning electron micrographs of examples for Fe nanostructures prepared by EBID (a) and EBISA (b) using $Fe(CO)_5$ as a precursor on native oxide on 100 nm silicon nitride membranes. In both cases, the irradiation step was performed using 15 keV electrons and a beam current of 400 pA. The EBID field deposit (a) was irradiated with a dose of 0.1 C/cm² while $Fe(CO)_5$ was dosed at 3.0×10^{-7} mbar background pressure (enhanced by a dosing needle in close proximity, the surface pressure is approx. 9×10^{-6} mbar, cf. main document). After the irradiation was finished, the precursor was dosed for additional 160 min. The line irradiations (1 µC/cm) for the EBISA array of 200 nm spaced parallel lines (b) was conducted under UHV conditions ($\sim 2 \times 10^{-10}$ mbar) before $Fe(CO)_5$ was introduced (conditions as described before) for 250 min.

In both cases, the (poly-)crystalline nature of the structures is apparent from the images. The cubic shape is an indication for bcc α-Fe crystallites. This characteristic appearance of the Fe nanostructures prepared by EBID or EBISA from $Fe(CO)_5$ is only observed after autocatalytic growth has occurred.
